# Advancing behavioural guidance systems to help conserve the European eel (*Anguilla anguilla*)

**DOI:** 10.1038/s41598-026-54768-7

**Published:** 2026-05-27

**Authors:** Mhairi Miller, Helen Hadlington, Sophie Minns, Suleiman M. Sharkh, Paul S. Kemp

**Affiliations:** International Centre for Ecohydraulics Research, Faculty of Engineering and Physical Sciences, Southampton Boldrewood Innovation Campus, University of Southampton, Southampton, SO16 7QF UK

**Keywords:** Anguilliform, behaviour, electric fields, guidance, pulsed direct current, Behavioural ecology, Freshwater ecology

## Abstract

This study investigated yellow (experiment 1) and silver-phase (experiment 2) European eel (*Anguilla anguilla*) behavioural response to pulsed direct current electric fields. Eel were offered a choice of two channels through which to pass under either: (1) a treatment condition, in which eel could select either an Electrified Channel (EC) or one in which the electric field was negligible (Non-Electrified Channel – NEC), or (2) a control in which the electric field was absent in both routes. In experiment 1, the influence of the EC field strength and direction of approach (upstream or downstream) on both *initial* and *total channel passage* and *avoidance* (*reaction*, *route change* and *rejection*) was assessed. In experiment 2, the influence of EC field strength and pulse frequency on *initial channel passage* and *avoidance* was investigated. The percentage of eel that passed the NEC under the treatment did not differ from the control in either experiment. In experiment 1, yellow-phase eel exhibited greater *total avoidance* in the EC when travelling upstream; but field strength had no effect. In experiment 2, silver-phase eel exhibited greater *initial avoidance* in the EC, but neither field strength nor frequency were influential. These findings will help optimise the design of devices that employ electric fields to guide eel.

## Introduction

The critically endangered European eel (*Anguilla anguilla*) population has declined by more than 90% over the past half-century^1,2^. Eel have been negatively impacted by multiple stressors that include overfishing^3^, habitat loss^4^, pollution^5^, shifts in oceanic currents^6^, non-native parasites^7^ and river infrastructure^8^. Moving fish can be impeded by man-made structures, such as dams and weirs, or entrained at water intakes, e.g. at hydropower and pumping stations^9–11^. Furthermore, compared to other species, eel may be at greater risk of injury and mortality, e.g. from blade strike and grinding during passage through turbines and pumps^12,13^ due to their elongated body morphology, leading in some cases to 100% mortality (e.g.^14^.

Concerns related to the migratory delay, injury and mortality of European eel at river infrastructure has led to the development of environmental impact mitigation technologies, such as bespoke eel passes^15–17^ and screens^18^. These have largely focused on the upstream migrating juveniles (glass eel and elver) (e.g.^19,20^. and downstream moving adults (silver-phase eel) (e.g.^21,22^), even though eel spend the vast majority of their life (e.g. more than two decades^23^, inhabiting the freshwater environment during their resident yellow-phase, during which they encounter river infrastructure during both upstream and downstream exploratory movements^24^. Nevertheless, knowledge gaps on the movement behaviour of this life stage remain (see^25,26^. To minimise the negative impact of river infrastructure on eel there is a need to consider the entire lifecycle, rather than concentrate only on the active migratory life stages. Therefore, greater understanding of the value of environmental impact mitigation strategies that target yellow-phase eel is also needed.

Physical and mechanical fish screens traditionally installed at water intakes can themselves negatively impact the target species they were designed to protect^13^. For example, eel become impinged and suffocate on the screen surface if the water velocities at the face exceed burst swimming capabilities, preventing escape (e.g.^27^. for silver-phase European eel). Further, whilst the efficiency of screens designed to guide silver-phase European eel to bypass routes can be high^28^ in other cases it was as low as 0%^29^, delaying migration and increasing energetic expense and predation risk^30,31^. To better protect eel at water intakes there is a need to reduce risks of impingement on screens and enhance the efficiency of guidance. Improved efficacy of physical fish screens may be achieved by integrating behavioural stimuli (e.g. light, bubbles, acoustics) that induce avoidance in the target species (e.g.^32–34^. To achieve this for eel, however, there is a need to identify the most appropriate cue(s) that may be used as a deterrent or as part of a combined physical-behavioural screening system.

A range of stimuli have been tested in efforts to identify those most appropriate for eel guidance. Light^35,36^ and acoustics^37,38,34^ have received much attention, with variable results reported. For example, underwater light was observed to deflect 80.6% of downstream migrating silver-phase eel in one study^35^, but only 50–65% in another^36^. In relation to acoustics, while infrasound was observed to effectively deflect adult eel in one study^39^, others found limited^37^ or even no response^40^ to similar frequencies. Other behavioural cues have received less attention, resulting in a lack of understanding and agreement on the most appropriate stimuli to deter and guide different life stages. There is a need to further test the effectiveness of other stimuli to identify the most appropriate cues that might be employed in a future integrated deterrent system.

Electric fields may offer potential value for fish guidance systems as they may produce a more consistent response than other stimuli^41^. While electricity has previously been used to deter fish, e.g. to control the upstream movements of invasive species (e.g., grass carp, *Ctenopharyngodon idella*^42^; sea lamprey, *Petromyzon marinus*^43^; Eurasian Ruffe, *Gymnocephalus cernuus*^44^ and common carp, *Cyprinus carpio*^45^, application to eel guidance has been limited (see^46–48^. In the case of conservation, as opposed to invasive species control, electric fields have been developed to protect migrating fish. The disadvantage of using electricity is that there is a risk it may stun, and in the worst cases injure or even kill, the target species it is designed to protect. Indirect mortality of an incapacitated fish may also result due to predation or because it is swept downstream into the hazardous area from which it should have been deflected, such as an intake to a hydropower turbine, with no means of escape^49^. For these reasons, electric deterrents tend to use graduated fields that reduce the risk of a fish being stunned^50^, and focus on upstream moving fish, e.g. at the tail race of hydropower stations^51^, that would be swept with the currents out of harms way even if incapacitated^52^. To further improve the potential and safety of electric fish guidance systems, research is needed to optimise field distribution and other parameters that are known to influence fish behaviour, such as pulse frequency^53^ for steelhead, *Oncorhynchus mykiss* and Pacific lamprey, *Entoshpenus tridentatus*) and field strength^54^ for silver carp, *Hypophthalmichthys molitrix*).

This study investigated the response of yellow- (experiment 1) and silver-phase (experiment 2) European eel to pulsed direct current (PDC) electric fields when offered a choice of route of passage via two channels of equal dimension in an experimental flume. The two channels provided an opportunity to select a route: (1) with either an electric field (Electrified Channel - EC) or where the influence of electricity was negligible (Non-Electrified Channel - NEC) under treatment conditions; or (2) a control in which the electric field was absent in both channels. In experiment 1, the influence of electric field strength and direction of approach on *initial*, and *total*, *channel passage* and *avoidance* were investigated. Conversely, in experiment 2, the influence of electric field strength and frequency on *initial channel passage* and *avoidance* was tested. Assuming that fish exhibit aversion to electricity, we predicted that eel avoidance would be:

(1) associated with the EC when offered a choice (experiments 1 and 2, Hypothesis 1 [H1]);

(2) positively related to field strength (experiment 1 and 2, Hypothesis 2 [H2]), as would *passage* through the NEC;

(3) more likely during upstream approach (experiment 1, Hypothesis 3 [H3]); and.

(4) greater under the higher frequency (10 Hz) condition (experiment 2, [H4]).

## Results

### Experiment 1: Yellow-phase eel

#### Channel passage

**Fig. 1:**
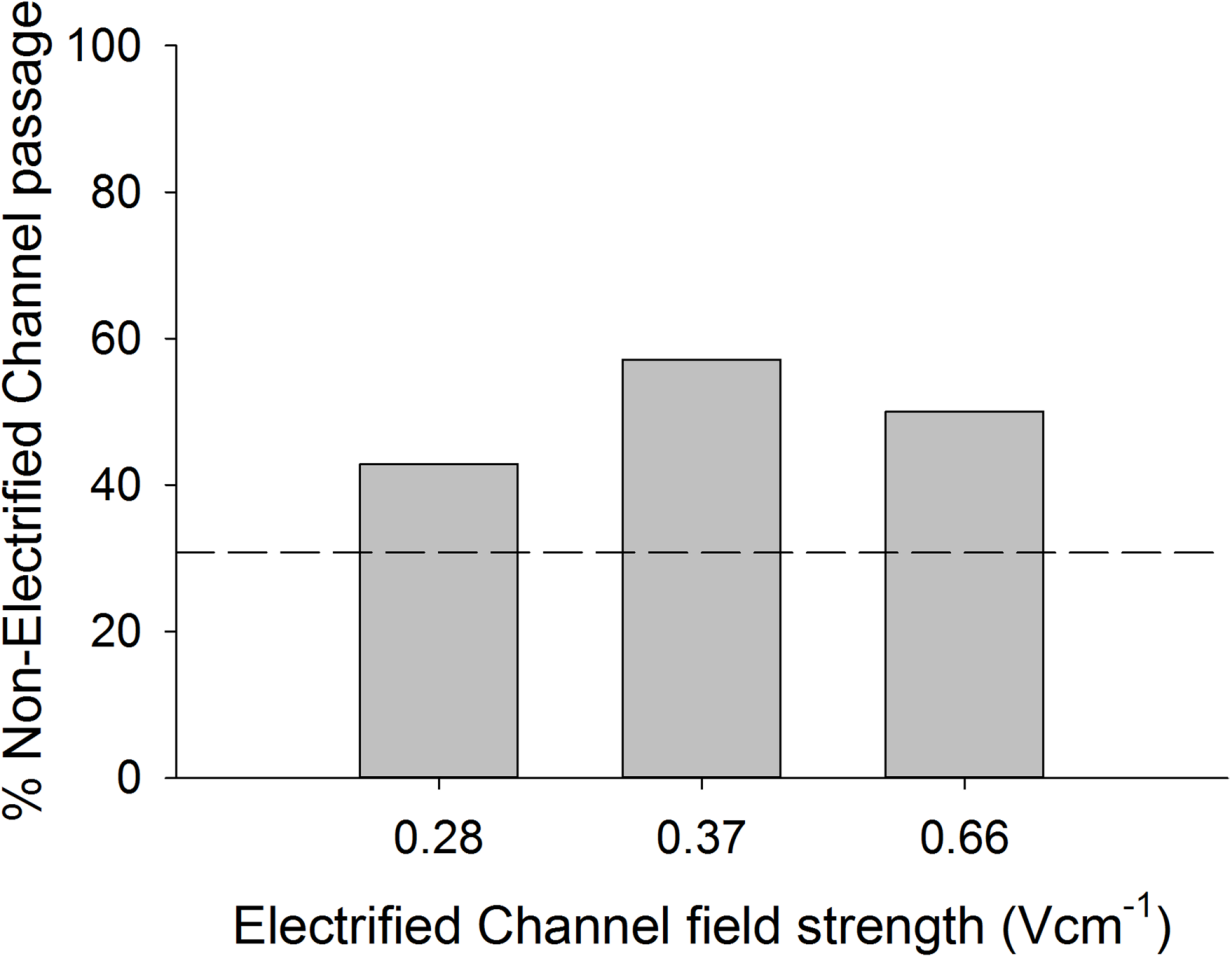
Percentage of yellow-phase European eel that initially passed the Non-Electrified Channel (NEC) under the three Electrified Channel (EC) field strengths (0.28, 0.37 and 0.66 Vcm^− 1^). Dashed line shows the percentage obtained under the control (0 Vcm^− 1^).

Although the percentage *initial channel passage* through the NEC was higher under the treatment conditions compared to the control, the difference was not significant (*p* > 0.05) (Fig. 1), contradicting H1. However, surprisingly passage under the control (0 Vcm^− 1^) was not equal between both channels (EC vs. NEC).

**Fig. 2:**
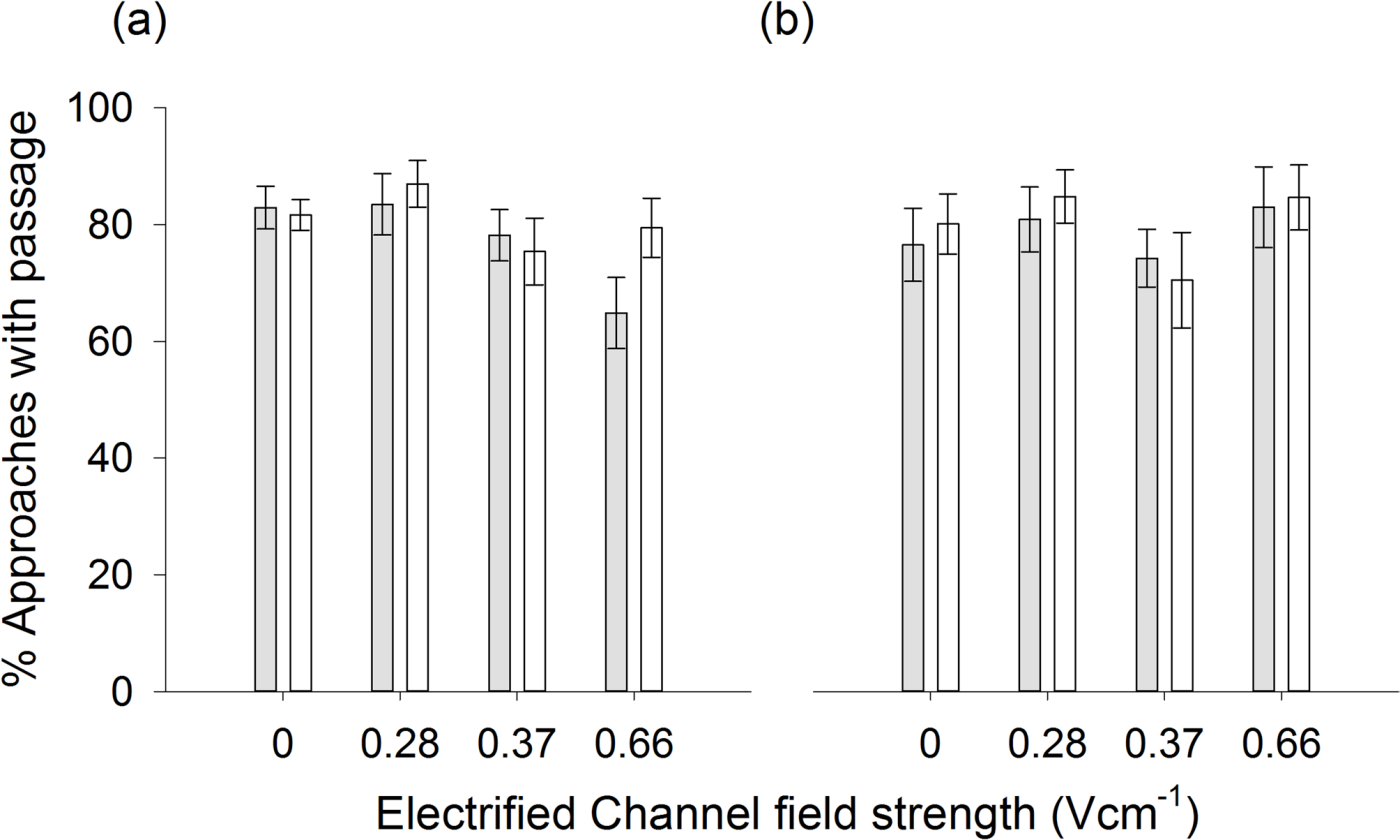
Mean [± SE] percentage *total passage* of (**a**) downstream and (**b**) upstream approaches by yellow-phase eel through the Electrified (EC) (grey bars) and Non-Electrified Channel (NEC) (white bars) under the control (0 Vcm^− 1^) and 0.28, 0.37 and 0.66 Vcm^− 1^ treatments during the trial.

The probability of eel passing as defined in terms of *total channel passage* was not influenced by channel (χ^2^(1) = 3.37, *p* = 0.07), field strength (χ^2^(3) = 5.3, *p* = 0.15), or direction of approach (χ^2^(1) = 1.06, *p* = 0.3), respectively contradicting H1, 2 and 3 (Fig. 2).

#### Avoidance behaviour

**Fig. 3:**
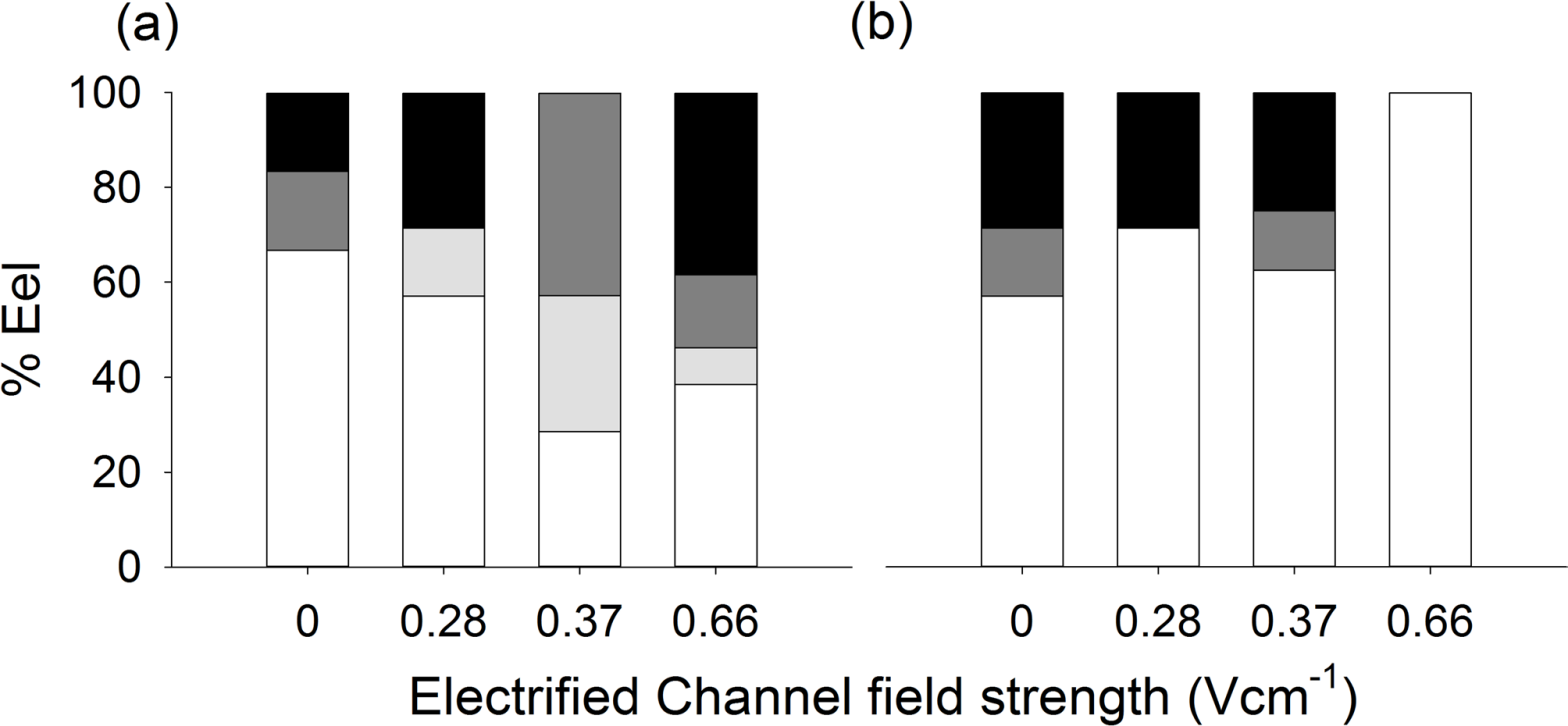
Percentage of yellow-phase European eel that exhibited *no change* (white), or an *initial avoidance* response as a *reaction* (light grey), *route change* (dark grey) or *rejection* (black) on encountering a choice of route through either (**a**) Electrified (EC) or (**b**) Non-Electrified (NEC) Channel under the control (0 Vcm^− 1^), 0.28, 0.37 and 0.66 Vcm^− 1^.

In contradiction to H2, there was no difference in *initial avoidance* between the EC and NEC (z = -1.76, *p* = 0.08) (Fig. 3). As a consequence, data were aggregated for comparison across field strengths, with no difference in the occurrence of *initial avoidance* observed between the EC and NEC channels (χ^2^(3) = 2.84, *p* = 0.42).

**Fig. 4:**
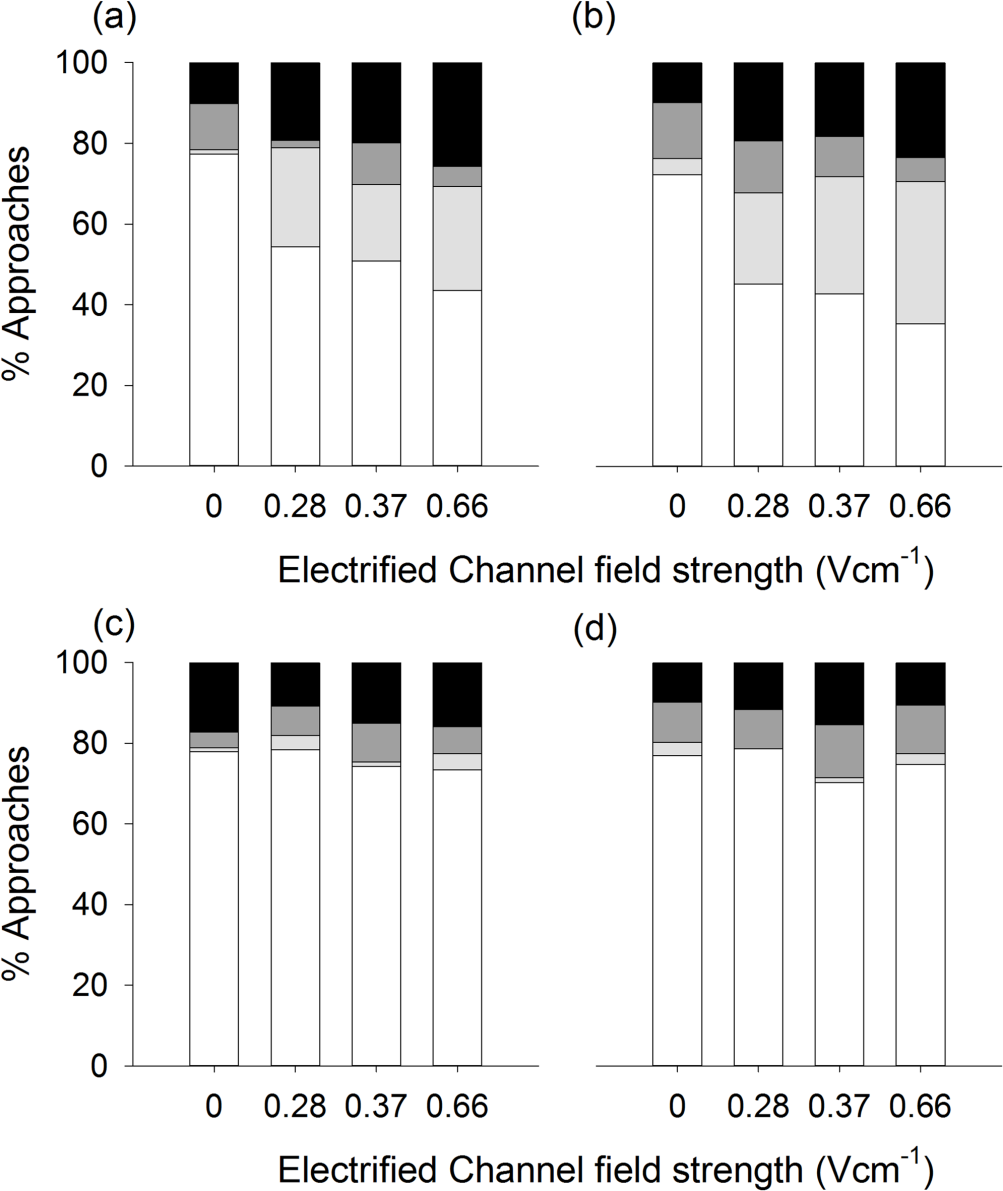
Percentage of downstream (**a**,** c**) and upstream (**b**,** d**) approaches of yellow-phase European eel in which either *no change* (white bars) or *total avoidance* was exhibited for the (**a**,** b**) Electrified (EC) and (**c**,** d**) Non-Electrified Channel (NEC) under the control (0 Vcm^− 1^) or 0.28, 0.37 and 0.66 Vcm^− 1^ treatments. Total avoidance was categorised as *reaction* (light grey), *route change* (dark grey) or *rejection* (black).

In support of H2, *total avoidance* was influenced by channel with greater *reaction* (χ^2^(1) = 88.6, *p* < 0.001), *rejection* (χ^2^(1) = 6.02, *p* = 0.014), and less *no change* (χ^2^(1) = 85.6, *p* < 0.001) observed in the EC (Fig. 4). In contrast, channel had no effect on the occurrence of *route change* (χ^2^(1) = 0.014, *p* = 0.91) throughout the trials.

Due to differences in occurrence of behaviours between channels, the effect of field strength and direction of approach was tested independently for each channel. In the EC, field strength effected the occurrence of *no change* (χ^2^(3) = 30.4, *p* < 0.001), *reaction* (χ^2^(3) = 29.4, *p* < 0.001) and *rejection* (χ^2^(3) = 9.91, *p* = 0.02), supporting H2 (Fig. 4a, b). *Reaction* and *no change* were respectively more and less common under the treatments than the control (*no change*: 0.28 Vcm^− 1^: z = − 3.83, *p* < 0.001, 0.37 Vcm^− 1^: z = − 4.31, *p* < 0.001 and 0.66 Vcm^− 1^: z = − 5.23, *p* < 0.001 and *reaction*: 0.28 Vcm^− 1^: z = 4.75, *p* < 0.001, 0.37 Vcm^− 1^: z = 4.85, *p* < 0.001 and 0.66 Vcm^− 1^: z = 5.38, *p* < 0.0001). *Rejection* occurred more frequently under the 0.66 Vcm^− 1^ treatment than the control (z = 3.13, *p* = 0.009). In contrast, field strength did not influence the occurrence of *route change* (χ^2^(3) = 7.33, *p* = 0.06). In the NEC, none of the avoidance behaviours were influenced by field strength in the adjacent EC (*no change*: χ^2^(3) = 2.06, *p* = 0.56, *reaction*: χ^2^(3) = 2.09, *p* = 0.55, *route change*: χ^2^(3) = 2.27, *p* = 0.52 and *rejection*: χ^2^(3) = 1.03, *p* = 0.79) demonstrating the weak electric field (i.e. leakage) in the NEC had no effect on behaviour (Fig. 4c, d).

**Fig. 5:**
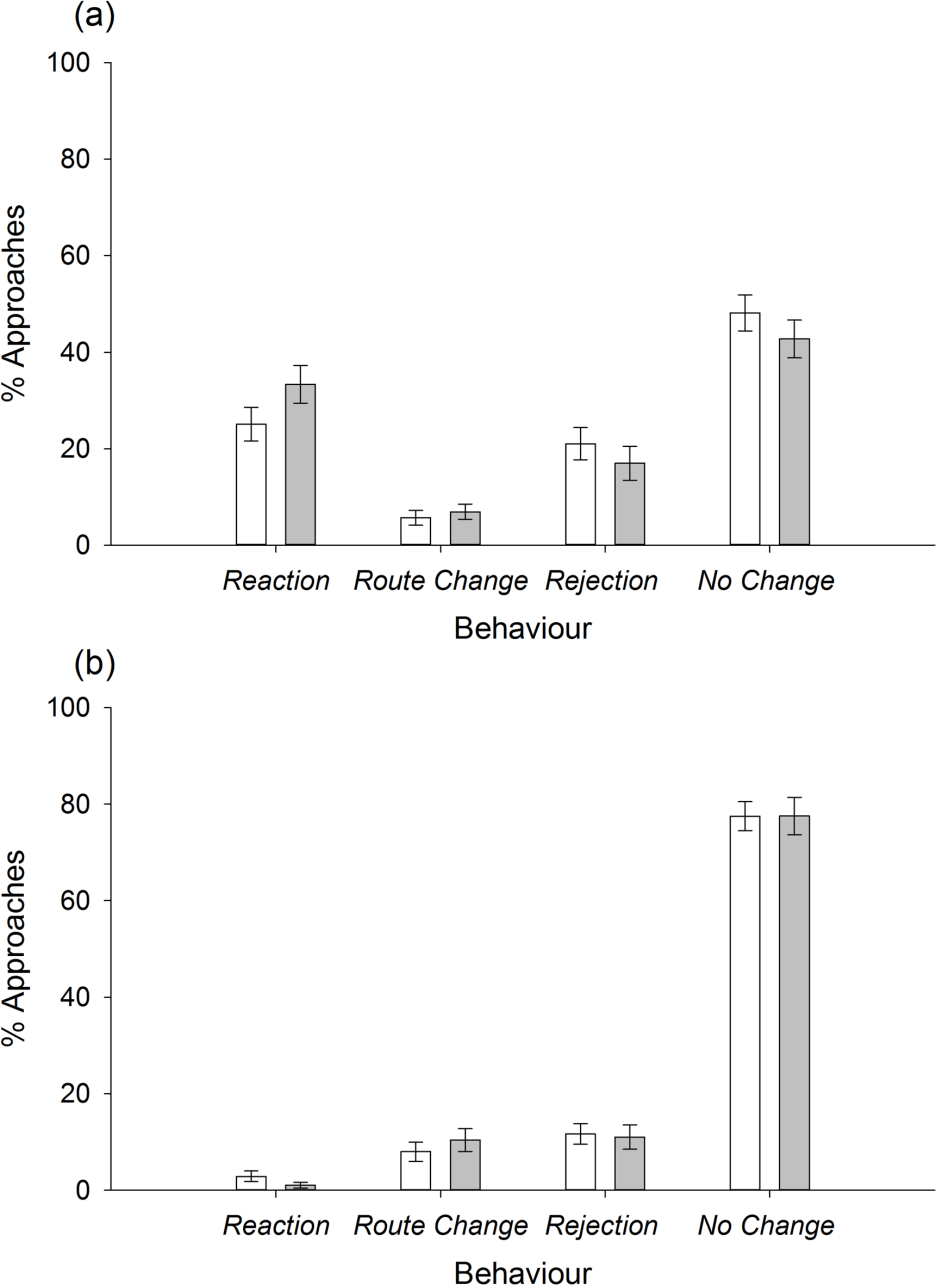
Mean percentage of approaches in which *no change* or *total avoidance* [± SE] was exhibited by yellow-phase European eel in an (**a**) Electrified (EC) and (**b**) Non-Electrified Channel (NEC) when moving downstream (white bars) or upstream (grey bars). Treatment data were aggregated for three field strengths (0.28, 0.37 and 0.66 Vcm^− 1^). Total avoidance is categorised into r*eaction*, *route change*, and *rejection*.

In support of H3, *no change* was observed less frequently for upstream than downstream approaches (χ^2^(1) = 5.38, *p* = 0.02) (Fig. 5a). In contrast, *reaction*, *route change* and *rejection* were not affected by direction of approach (*reaction*: χ^2^(1) = 3.63, *p* = 0.06; *route change*: χ^2^(1) = 3.32, *p* = 0.07; and *rejection*: χ^2^(1) = 0.15, *p* = 0.7), and direction of approach did not influence the occurrence of *total avoidance* in the NEC (*no change*: χ^2^(1) = 0.02, *p* = 0.89; *reaction*: χ^2^(1) = 1.7, *p* = 0.19; *route change*: χ^2^(1) = 1.75, *p* = 0.19; and *rejection*: χ^2^(1) = 0.21, *p* = 0.64) (Fig. 5b).

### Experiment 2: Silver-phase eel

#### Channel passage

**Fig. 6:**
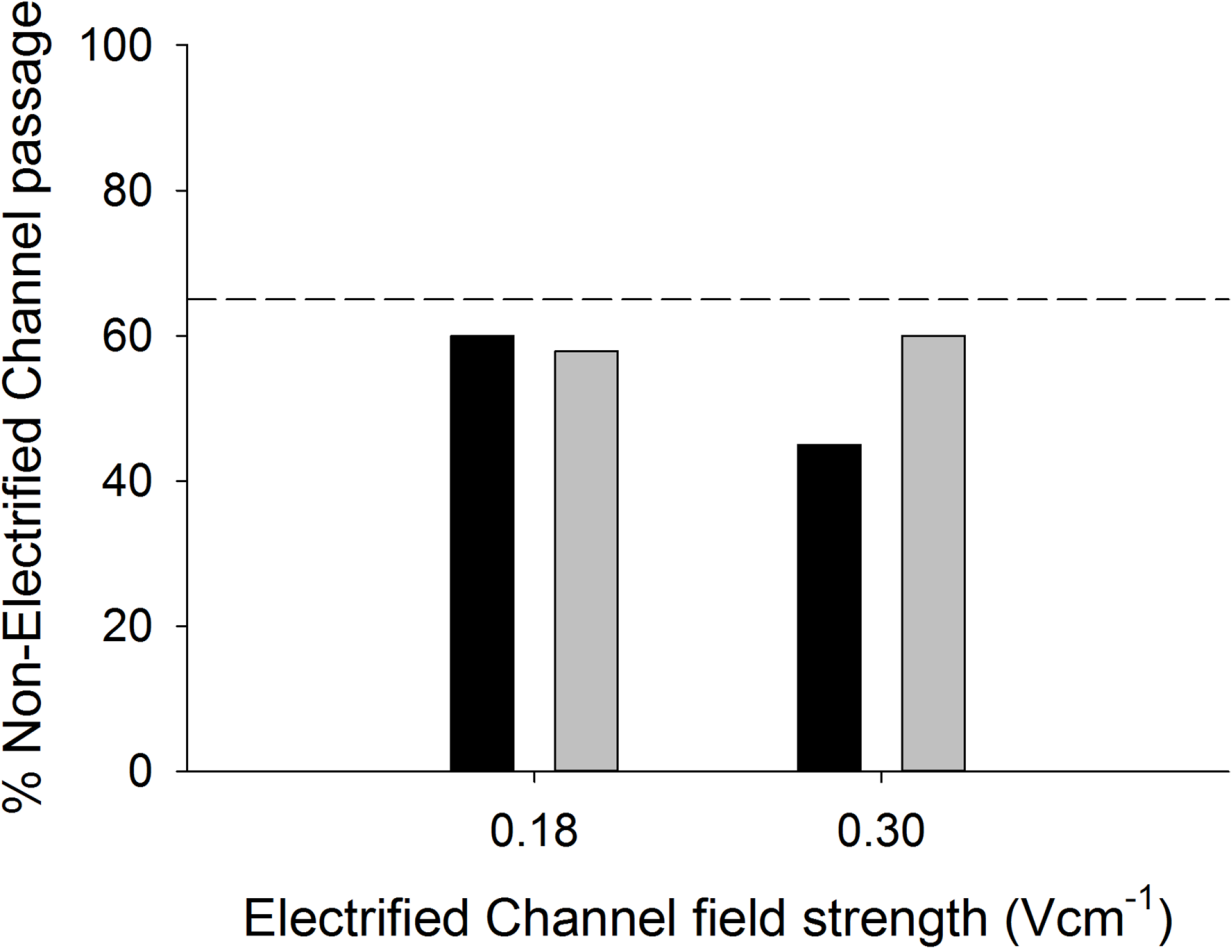
Percentage of silver-phase European eel that initially passed the Non-Electrified Channel (NEC) under the two Electrified Channel (EC) field strength treatments (0.18 and 0.30 Vcm^− 1^) under 2 Hz (black bars) and 10 Hz (grey bars). The dashed line indicates the percentage obtained under the control (0 Vcm^− 1^).

In contradiction to H1, there was no difference between the percentage *initial channel passage* through the NEC under the control and any of the treatments (*p* > 0.05) (Fig. 6). However, passage under the control was not equal between both channels as would be expected.

#### Avoidance behaviour

**Fig. 7:**
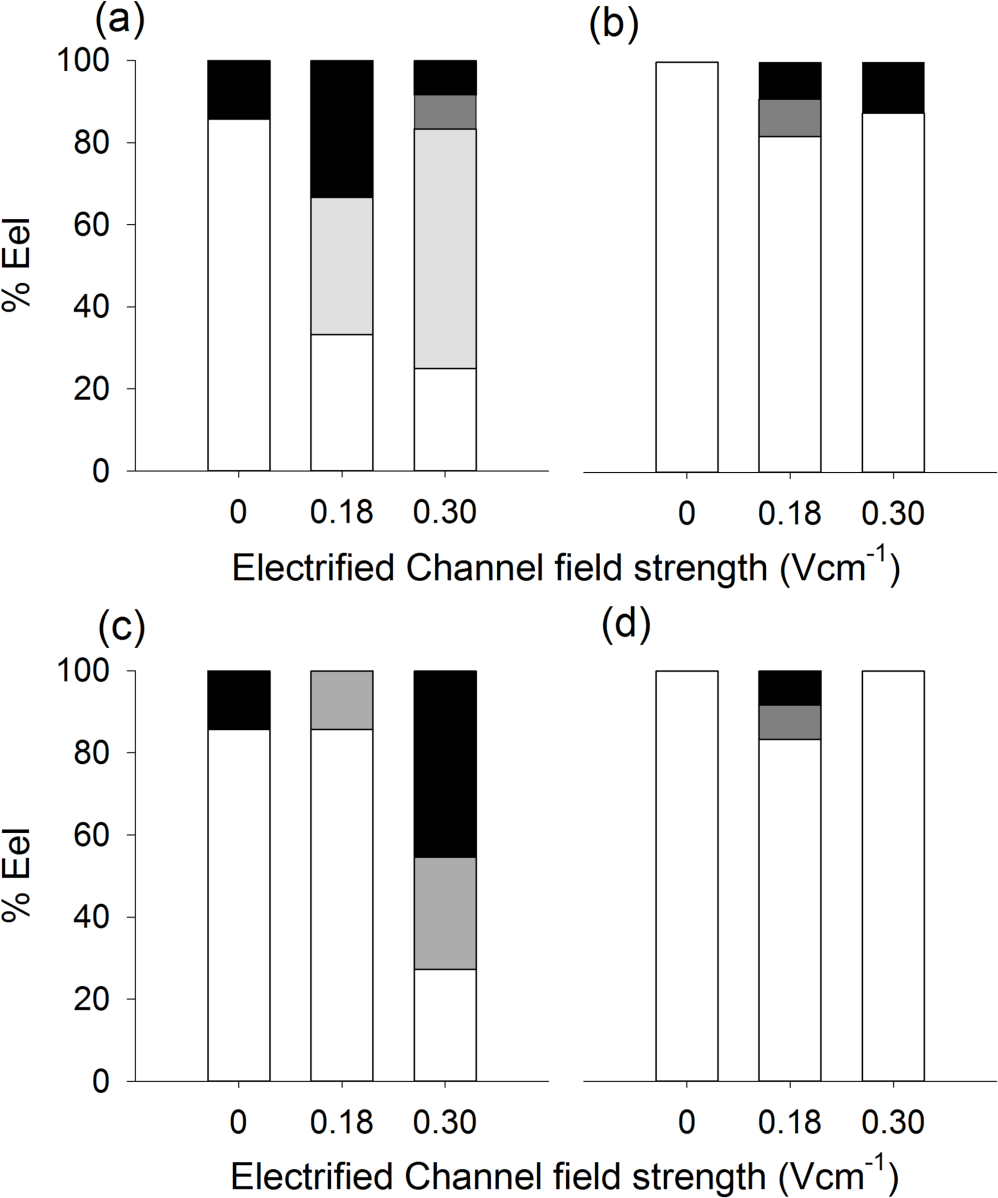
Percentage of downstream migrating silver eel that exhibited *initial avoidance* in either (**a**,** b**) 2 or (**c**,** d**) 10 Hz PDC electric fields as they encountered a choice of channel through either an (**a**,** c**) Electrified (EC) or (**b**,** d**) Non-Electrified Channel (NEC) under two treatment field strengths (0.18 and 0.30 Vcm^− 1^) and a control (0 Vcm^− 1^). White, light grey, dark grey and black represent *no change*, *reaction*, *route change* and *rejection*, respectively.

In support of H2, channel influenced the occurrence of *initial avoidance* with more (61.5%) eel exhibiting a response in the EC than the NEC (12.5%) (z = − 4.39, *p* < 0.001) (Fig. 7).

Due to the differences in *initial avoidance* between channels (EC vs. NEC), the data were separated for comparison between field strengths and frequency. In the EC, field strength influenced the occurrence of *initial avoidance* (χ^2^(2) = 9.35, *p* = 0.009) (Fig. 7a, c) with greater avoidance under the 0.30 Vcm^− 1^ than the control (z = 2.4, *p* = 0.04) (supporting H2). In contradiction to H4, frequency (2 vs. 10 Hz) did not affect the occurrence of *initial avoidance* in the EC (χ^2^(1) = 1.89, *p* = 0.17). In the NEC, neither field strength nor frequency influenced the occurrence of *initial avoidance* (field strength: χ^2^(2) = 4.26, *p* = 0.12 and frequency: χ^2^(1) = 1.58, *p* = 0.21) (Fig. 7b, d).

## Discussion

Both yellow- and silver-phase European eel exhibited avoidance when exposed to electric fields. In contrast to our predictions, however, neither phase preferred to pass the Non-Electrified Channel under any of the treatment conditions presented. In experiment 1, *total avoidance* was more often observed for yellow-phase eel in the Electrified Channel and during upstream approaches, in support of our hypotheses. In experiment 2, also as predicted, silver-phase eel exhibited more frequent *initial avoidance* in the Electrified than Non-Electrified Channel, although field strength and pulse frequency were not influential. The results of this study highlight the potential for the use of electric fields to elicit avoidance, although both yellow and silver-phase European eel demonstrated an ability to tolerate the stimulus and persist on their passage trajectory. Continued optimisation of electric deterrence is needed to enhance guidance further.

In contradiction of our first hypothesis, yellow-phase eel did not prefer to pass through the Non-Electrified Channel under the treatments any more than the control (experiment 1). The ability of eel to tolerate the electric field encountered is likely because the field strengths and/or frequencies used were insufficient to change the swimming trajectory away from the Electrified Channel under the conditions created. Our selection of field strengths were based on previous experience of using silver-phase eel^47^ and ethical considerations. However, higher field strengths may be required for the smaller yellow-phase eel that are likely to better tolerate the test fields due to the relationship between body length and electrosensitivity, with shorter fish being less sensitive due to the lower voltage gradient measured along the length of their body^55^. Indeed, the effectiveness of electric fields for fish guidance has previously been linked to body size, with lower efficiencies observed for smaller fish (e.g.^56^. for rainbow trout, *Oncorhynchus mykiss*).

As for experiment 1, silver-phase eel did not prefer to pass through the Non-Electrified Channel in experiment 2, in contradiction to our prediction. This may have been a consequence of the spatial variation in distribution of the electric field, with the greatest relative difference in field strengths between the two routes occurring within the channels themselves, rather than that encountered on approach. If the relative difference in field strengths on approach was higher, providing a sufficient gradient, eel may have been better able to differentiate between the two routes and exhibit a clearer choice for areas of weaker field strength prior to committing to entering one of the channels^57^. Without an opportunity for eel to sample an alternative route and deviate course when encountering the strongest electric field, they tended to remain on the same trajectory having entered a channel. As field strengths were insufficient to elicit *rejection*, eel did not swim back upstream and pass the alternative channel instead. Interestingly, eel did not exhibit an expected 50:50 channel passage under the control in either experiment. While the reason for this remains unknown, it may relate to some unmeasured characteristics related to the flume, such as the hydrodynamic conditions created. The alternation of treatments between the two channels ensured such unknown confounding variables were controlled for.

*Initial avoidance* was more common in the Electrified Channel for silver-phase eel, in support of our second hypothesis. This was not so for yellow-phase eel for which no difference was observed between the treatment field strengths and control. The apparent difference between the life-stage may be explained by a difference in the characteristics of electric field generated in the two experiments; the field was more localised and restricted to the Electrified Channel in experiment 2. When considering *total avoidance*, however, yellow-phase eel appeared to discriminate between the two channels, with more frequent *reaction* and *rejection* in the Electrified Channel and more frequent *reaction* and less *no change* under treatment conditions than the control. Furthermore, compared to the control there was greater *rejection* under the highest field strength (0.66 Vcm^− 1^), although in contradiction to the second hypothesis there was no relationship between field strength and *total avoidance*. In experiment 2, while silver-phase eel exhibited more frequent *initial avoidance* in response to the highest field strength (0.30 Vcm^− 1^) in the Electrified Channel compared to the control, there was no difference between treatments (i.e. 0.18 vs. 0.30 Vcm^− 1^), presumably because the relative difference in field strength was insufficient to effect avoidance behaviour. More work is needed to determine whether relative difference in voltage influences avoidance exhibited by eel.

In support of hypothesis 3, the direction of approach (upstream or downstream) influenced *total avoidance* in yellow-phase eel with less avoidance observed when eel moved downstream. The use of electric fields for behavioural guidance is thought to be more effective for upstream moving fish^52^ because the direction of flow will ensure fish are displaced downstream with minimal effort if they detect the electrified zone and select to avoid it. In contrast, fish that are moving downstream are likely to be travelling faster with less time to respond so may be swept with the flow through the electrified area should the field be below some threshold at which eel respond.

Pulse frequency (2 and 10 Hz) had no effect on *initial channel passage* or *initial avoidance* for silver-phase eel (experiment 2) contradicting the fourth and final prediction. In the context of electric fishing, higher pulse frequencies can cause more adverse responses (i.e. myoclonic jerks)^58^ and greater avoidance (e.g.^53^. for steelhead trout and Pacific lamprey). It is unclear why we observed a lack of response in this study although interspecific variation and our focus on a downstream moving fish may explain the apparent lack of sensitivity to differences in frequency.

This study should inform the future direction of the design of electric field guidance for European eel. Both life-stages considered here exhibited avoidance when exposed to electric fields, but this did not translate to an ability to manipulate their trajectory, likely due to a restriction on eel movement in the lateral dimension as a result of the laboratory experimental set-up or due to insufficient sample sizes. This study demonstrates the trade-off in design in which on one hand a sufficiently strong electric field is needed to induce avoidance and guide eel, while on the other hand a need not to cause undue stress, or in the worst cases incapacitation and injury. This is a key challenge in the design of electric guidance systems, the influence of which is likely to vary with multiple factors, including species, life-stage, body length, prior experience, motivational status and site-specific context (e.g. water velocity and conductivity). Certainly, future behavioural guidance studies should confirm long term eel response under more realistic field settings in which fish are not spatially constrained by physical structures as in this study and assess potential stimuli desensitisation. Moreover, understanding stimulus intensity and coverage for proposed field sites will help bridge the gap between controlled laboratory studies and real-life applications^59^. Nevertheless, future fine-scale laboratory research might focus on optimising electrode orientation and electric field parameters (i.e. field strength and pulse frequency) to enhance guidance. Furthermore, the use of electric fields in combination with physical screens may enhance overall performance of guidance devices that attempt to manipulate behaviours of multiple species of fish.

## Methods

### Ethical note

All methods were followed in accordance with the UK national regulatory Animal Welfare Ethical Review Body’s (AWERB) guidelines and regulations. This study was approved by the University of Southampton’s Ethics and Research Governance Office (Ethics ID 51974) and reported in accordance with ARRIVE guidelines.

### Experimental set-up

**Fig. 8:**
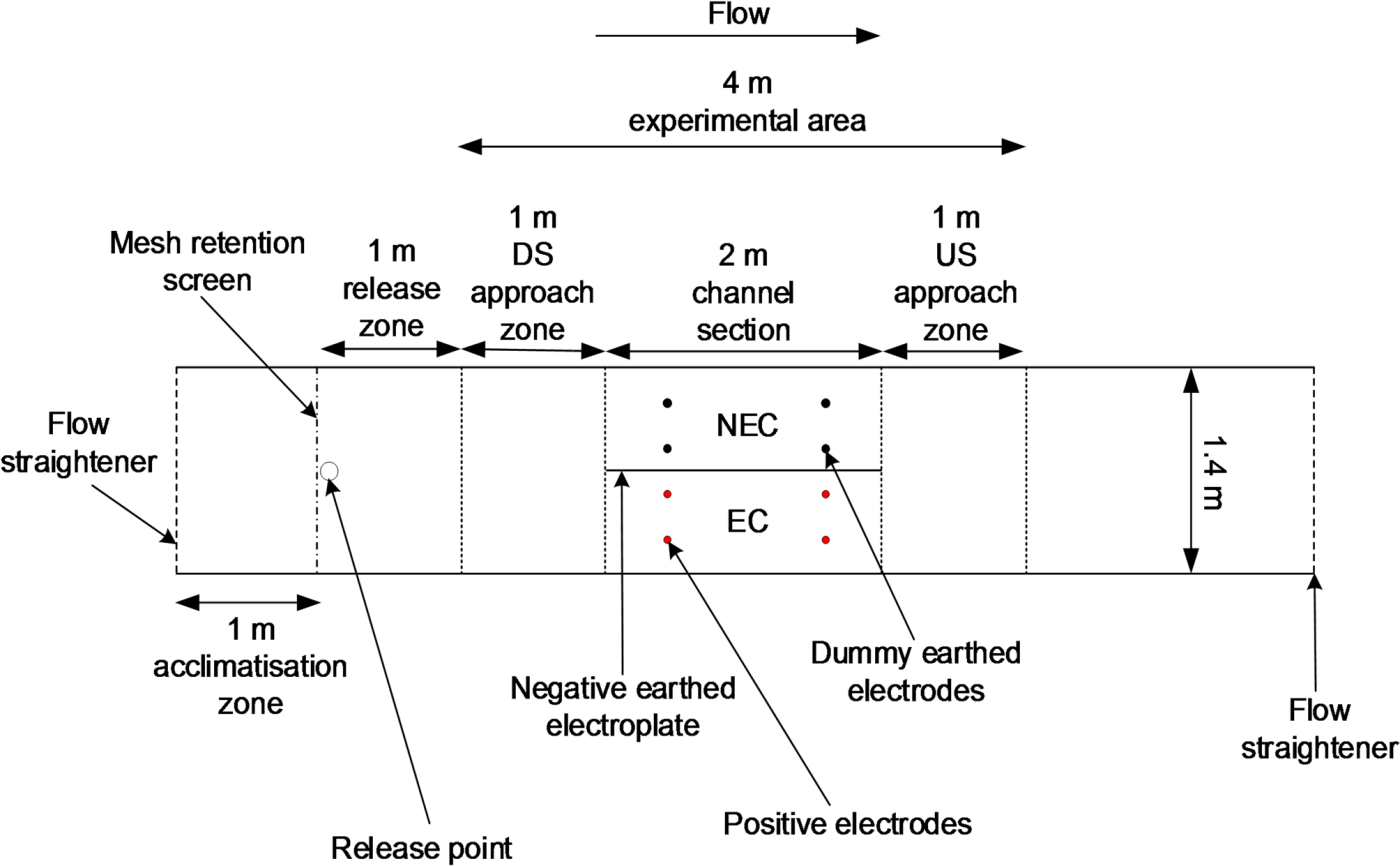
Plan of the set-up of an experiment to investigate European eel response to electric fields and subsequent channel passage in an open channel flume. The flume was divided longitudinally down the centre of a 2 m long section of the channel by a negative earthed aluminium sheet (electroplate). Two sets of two positive electrodes were installed to create the Electrified Channel (EC). An adjacent Non-Electrified Channel (NEC) was created by installing two sets of two earthed electrodes (dummy) at the same longitudinal position as the positive electrodes in the EC. Note the yellow-phase eel trials (experiment 1) had an additional 1 m between the release point and experimental area.

Experiments 1 and 2 were conducted in an indoor open channel flume (21.4 m long, 1.4 m wide and 0.6 m deep) at the International Centre for Ecohydraulics Research (ICER) facility, University of Southampton. A negative earthed aluminium sheet (200 cm long x 76.3 cm wide x 0.5 cm deep) was used to divide the flume longitudinally into two channels of equal dimension that under the treatment conditions were defined as either the Electrified (EC) or Non-Electrified Channel (NEC) (Fig. 8). In the EC, two sets of two positive stainless-steel electrodes (80 cm long x 1 cm diameter) were installed across the channel width at 27.6 cm apart. In the NEC two sets of two dummy earthed electrodes were located at the same positions as the positive electrodes in the EC. Earthed electrodes and the aluminium sheet were arranged to best localise the electric field within the EC and prevent it extending outside the experimental area. Each 80 cm long electrode was maintained 1 cm above the flume floor and covered with electrically insulated mesh fabric (mesh size: 0.28 × 0.79 mm) to prevent direct contact between the eel and live electrodes.

The external glass walls of the flume were covered in a black screen to prevent disturbance to the fish by the observer. Overhead cameras (experiment 1: 8 SWANN CCTV 720p and experiment 2: 6 SWANN 780 and 1080p) and infrared lights (780–850 nm wavelength, experiment 1: 9 and experiment 2: 4 units) were used to provide sufficient illumination for video capture under conditions of darkness.

Treatment field strengths (Table 1) were generated using a Smith-Root Electrofishing pulse generator (BP-1.5 POW) and selected considering the results of a previous study in which silver-phase eel exhibited avoidance under field strengths of 0.15–0.3 Vcm^− 1^ in flowing water^47^. The slightly higher field strengths tested in experiment 1 were based on the shorter body length of yellow-phase eel (yellow: 24–55.5 cm; silver: 31–67 cm) as body length is a predictor of electrosensitivity^55,60^. Although a weak electric field was present in the NEC due to leakage (mean field strength ≤ 0.081 Vcm^− 1^) it was deemed negligible for the purpose of this study as previous research on adult silver-phase eel indicated that the mean threshold field strength required to elicit a response was 0.10 Vcm^− 1^ (2 Hz, 100 ms waveform) and 0.11 Vcm^− 1^ (10 Hz, 10 ms waveform)^47^.

**Fig. 9:**
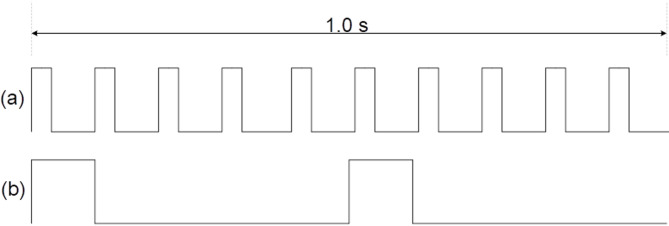
PDC frequencies: (a: 10 Hz; b: 2 Hz) used to investigate European eel response to a two-choice test electric field. The 10 Hz frequency was used for yellow-phase eel (experiment 1) and both frequencies were tested in silver-phase eel trials (experiment 2).

Waveforms were selected accounting for ethical considerations as PDC frequencies < 15 Hz reduce probability of injury in eel^61^. As both 2 and 10 Hz pulse frequencies have been observed to effectively guide fish (e.g. 2 Hz: for juvenile European eel^62^, ; 10 Hz: sea lamprey^43^, a PDC waveform with a frequency of 10 Hz and 20 ms pulse width was used (Fig. 9a) in experiment 1 (yellow-phase eel), and 2 Hz (100 ms pulse width) and 10 Hz (20 ms pulse width) waveforms (Fig. 9a, b) in experiment 2 (silver-phase eel).

**Table 1:** The characteristics of the electric field generated in an Electrified Channel (EC) and adjacent Non-Electrified Channel (NEC) in an experimental flume study to investigate yellow- and silver-phase European eel response to electricity (Experiments 1 and 2, respectively). The mean [± SE] and range field strengths were calculated throughout the experimental area. The number of replicates (n) for each treatment is provided. Note the EC and NEC field strengths were equivalent under 2–10 Hz (experiment 2).

Pulse Generator Output (V)	Electrified Channel (EC) Field Strength (Vcm^− 1^)		Non-Electrified Channel (NEC) Field Strength (Vcm^− 1^)		Sample Size (*n*)	Experiment
	Mean [± SE]	Range	Mean [± SE]	Range		
0 (Control)	0	0	0	0	13	1
10	0.28 [± 0.014]	0–0.74	0.02 [± 0.002]	0–0.11	14	1
12.5	0.37 [± 0.02]	0–1.48	0.035 [± 0.004]	0–0.30	15	1
15	0.66 [± 0.03]	0–2.04	0.081 [± 0.008]	0–0.93	14	1
0 (Control)	0	0	0	0	20	2
5	0.18 [± 0.011]	0–0.52	0.0012 [± 0.0004]	0–0.07	2 Hz: 20 10 Hz: 19	2
10	0.30 [± 0.014]	0–0.74	0.006 [± 0.001]	0–0.09	2 Hz: 20 10 Hz: 20	2

**Fig. 10:**
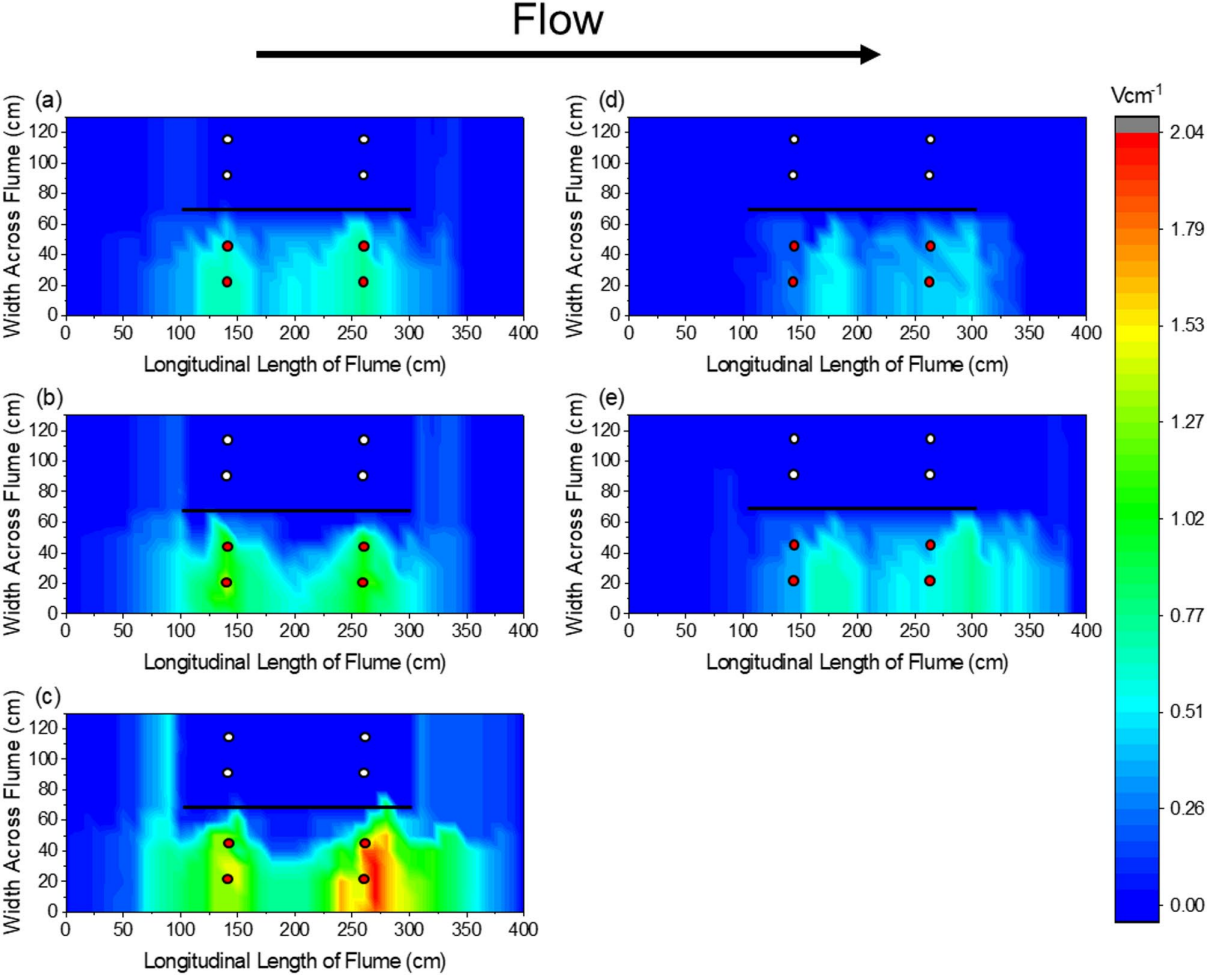
Electric field (Vcm^− 1^) generated by the pulse generator output for experiment 1: yellow-phase eel (a - c) of (a) 10 V (mean Electrified Channel [EC] field strength: 0.28 Vcm^− 1^), (b) 12.5 V (mean EC field strength: 0.37 Vcm^− 1^) and (c) 15 V (mean EC field strength: 0.66 Vcm^− 1^), and for Experiment 2: silver-phase eel (d - e) under (d) 5 V (mean EC field strength: 0.18 Vcm^− 1^) and (e) 10 V (mean EC field strength: 0.30 Vcm^− 1^). For experiment 2, field distribution was the same for both 2 and 10 Hz so only one map is depicted. The *\:x* axes represent the longitudinal distance along the flume and the *\:y* axes the width of the flume. The negative earthed sheet (black line) extended from *\:x* = 100 to 300 cm and *\:y* = 69 cm. Positive electrodes (red dots) were positioned at *\:x* = 140 cm and *\:y* = 23 and 46 cm. The second set of positive electrodes were positioned at *\:x* = 260 cm and *\:y* = 23 and 46 cm with dummy earthed electrodes (white dots) at *\:x* = 140 and 260 cm and *\:y* = 92 and 115 cm. Measurements were taken at 15 cm water depth from the surface. Note the maximum electric field was parallel to the flow direction.

The electric field was mapped using a potential probe comprising of two-point conductors positioned 27 mm apart and connected to an oscilloscope (Gwinstek GDS-1052-U) via a differential probe module (Probemaster 4232). Measurements were taken in a grid at a spacing of 10 cm in the *\:x* and $\:y\:$directions at a water depth of 15 cm (from the water surface) to record peak-to-peak voltage (Fig. 10). Field strength was calculated as the quotient of the peak-to-peak voltage and the distance between the two-point conductors. Maps of the electric field were created for all EC treatment field strengths, under both the 2 and 10 Hz pulse frequencies (experiment 2 only) and the control (0 V) (Fig. 10). Water conductivity was 630 µScm^− 1^.

### Fish husbandry

European yellow-phase eel (n = 76) were obtained on 20 August 2019 by electric fishing at Bosham stream (50°50’44.1"N, 0°50’48.3"W), which flows into Chichester Harbour, West Sussex (UK). Adult European silver-phase eel (n = 106) were caught by a commercial fisherman using fyke nets installed in a drainage channel (52°30’ 2.55”N, 0°12’0.950”E) in the Lincolnshire Fens (UK) (7 and 20 November 2019). Eel were transported to the ICER facility at the University of Southampton (50°57′42.6″N, 1°25′26.9″W) in transportation tanks containing aerated river water. The fish were sorted into four 3000 L outdoor holding tanks and three 1500 L indoor holding tanks to ensure equal densities. The tanks were fitted with gravity fed external filters and UV filtration systems. A venturi pump on the filter outlets provided additional aeration to supplement that provided by large capacity air pumps. Fish health, water temperature (Mean Holding Tank Temperature [± SD]: Experiment 1 = 20.7 [± 1.18]°C, Experiment 2 = 13.3 [± 0.79]°C), and water quality were monitored daily, the latter ensuring high quality was maintained (pH: 7.8–8.4, Ammonia: 0 ppm, Nitrite: 0 ppm, Nitrate: < 40 ppm).

### Experimental procedure

Due to their natural life cycle and availability, experiments using yellow-phase (experiment 1) and silver-phase (experiment 2) eel were performed between 21 and 31 August 2019 and 25 and 29 November 2019, respectively. Trials were conducted between 18:00–06:00 to replicate natural nocturnal behaviour^63^ and treatments were alternated between trials. Ambient light intensities during tests were less than 0.01 lx.

A single eel was removed from the holding tank and placed in a plastic tube secured at both ends with mesh coverings before being positioned within the 1 m long acclimatisation zone, located upstream of the experimental area (Fig. 8). Eel were allowed a minimum of 60 min to acclimatise before being released facing downstream centrally, by removing the mesh at one end of the tube, immediately downstream of the mesh retention screen (release point, Fig. 8). For experiment 1 (yellow-phase eel), trials lasted 60 min allowing for repeated approaches in both the upstream and downstream directions. For experiment 2, due to the strong negative rheotaxis of silver-phase eel^63^, trials lasted a maximum of 60 min or until eel had passed downstream through the experimental area once. Each eel was used once only and weighed and measured at the end of each trial (Table 2).

Water depth and velocities were measured (Valeport Model 801) upstream and downstream of the 2 m long channel section (Fig. 8) every five trials (Table 2). The velocities were measured midway in the water column and at three lateral points across the flume width. Flume temperature and conductivity were measured at the start of each trial.

**Table 2:** Mean [± SD] measured variables during experimental trials of both yellow- and silver-phase European eel, *Anguilla anguilla* on encountering a two-choice electric field test in a flume.

Experiment	Mean Water Velocity [± SD] (ms^− 1^)	Mean Water Depth [± SD] (cm)	Mean Flume Temperature [± SD] (°C)	Mean Water Conductivity [± SD] (µScm^− 1^)	Mean Mass [± SD] (g)	Mean Total Length [± SD] (cm)
(1) Yellow-phase	Upstream: 0.203 [± 0.015] Downstream: 0.215 [± 0.013]	Upstream: 27.7 [± 1.1] Downstream: = 26.2 [± 1.2]	21.6 [± 1.59]	646.8 [± 6.36]	136.4 [± 63.1]	42.1 [± 7.77]
(2) Silver-phase	Upstream: 0.252 [± 0.005] Downstream: 0.260 [± 0.006]	Upstream: 30.4 [± 0.42] Downstream: 29.2 [± 0.61]	12.09 [± 0.55]	647.5 [± 4.81]	263.3 [± 125.6]	49.8 [± 9.60]

### Fish behaviour

To test our four experimental hypotheses (Table 3) video recordings were analysed to characterise and quantify channel passage and avoidance behaviour (Table 4) as eel passed through the experimental area (Fig. 8). Behaviour was recorded throughout the trial, and for eel that exhibited more than one response type that of the highest magnitude (1–4) was assigned (hierarchy of response adapted from^64^, Table 4).

**Table 3:** Summary of hypotheses tested and associated metrics and behaviours analysed for yellow- and silver-phase eel (experiment 1 and 2, respectively).

Hypothesis	Prediction	Metric	Behaviour	Experiment
H1	Avoidance will be associated with the EC when offered a choice	*Initial channel passage* *Total channel passage*	n/a n/a	1, 2 1
H2	Avoidance will increase with electric field strength; passage through NEC will increase	*Initial avoidance* *Total avoidance*	*Rejection* *Route Change* *Reaction* *No Change* *Rejection* *Route Change* *Reaction* *No Change*	1, 2 1
H3	Avoidance will be greater during upstream approaches	*Total avoidance*	*Rejection* *Route Change* *Reaction* *No Change*	1
H4	Avoidance will be greater under the higher frequency (10 Hz) condition	*Initial avoidance*	*Rejection* *Route Change* *Reaction* *No Change*	2

**Table 4:** Definitions of different categories of avoidance behaviours and metrics obtained for yellow- and silver-phase eel (experiment 1 and 2, respectively) on encountering a two-choice electric field test in a flume. Behaviours are ranked in a hierarchy of magnitude from *Rejection* (highest) to *No change* (lowest). (adapted from^64^.

Behaviour/Metric	Definition	Experiment
Behaviour		
*Rejection*	180⁰ turn in body position and movement for at least one body length	1 and 2
*Route Change*	Switching from one channel to the other (Electrified/Non-Electrified)	1 and 2
*Reaction*	Change in behaviour such as increase in swimming speed, recoil or contraction of the body	1 and 2
*No Change*	No change in swimming speed or body orientation	1 and 2
Metric		
*Initial Channel Passage*	Channel (EC or NEC) passed on first approach	1 and 2
*Total Channel Passage*	Percentage of total approaches with passage	1
*Initial Avoidance*	Behaviour exhibited on first approach	1 and 2
*Total Avoidance*	Percentage of approaches with behaviour	1

### Statistical analysis

Two of the yellow-phase and seven of the silver-phase eel did not approach the experimental area during the 60 min trial and were excluded from further analysis. Statistical analyses were conducted using R 4.0.2^65^. Deviation from normality was assessed using the Shapiro-Wilk test. Attempts were made to transform non-parametric data, and if unsuccessful, non-parametric tests were performed. Due to the low number of observations of some of the defined behaviours (*reaction*,* route change* and *rejection)* for *initial avoidance* in both experiments, they were combined as a single response. For statistical purposes the EC assigned during the treatment was also designated as such for the control.

In experiment 1, due to the small sample size of yellow-phase eel, a Fisher’s Exact test was used to determine whether *initial channel passage* for any of the treatments deviated from the percentage obtained under the control condition. *Initial avoidance* (Y/N) was assessed using a general linear model with binomial distribution. Main effects included were channel and field strength. *Total channel passage* (Y/N) and *total avoidance* (Y/N) were assessed using a generalised linear mixed model fitted with a binomial distribution with Eel-ID as a random effect to account for multiple approaches and passes by individual eel. Main effects included were channel selected, field strength and direction of approach (downstream vs. upstream).

In experiment 2, a goodness-of-fit (χ^2^) test determined whether *initial channel passage* (EC or NEC) by silver-phase eel for any of the treatments deviated from the percentage obtained under the control. A general linear model with binomial distribution was used to assess *initial avoidance* (Y/N). Main effects included were channel selected, field strength and frequency.

## Data Availability

Data and code published in this article are available from the University of Southampton repository at DOI: https://doi.org/10.5258/SOTON/D2529.
